# Nonsurgical endodontic treatment of type II dens invaginatus

**DOI:** 10.4103/0972-0707.55622

**Published:** 2009

**Authors:** Rajini MA, Anjali Kaiwar, Meena N, Anitha Kumari R, Ashish Shetty, Naveen DN, Shubhashini N

**Affiliations:** Department of Conservative Dentistry and Endodontics, V. S. Dental College and Hospital, K.R. Road, V. V. Puram, Bangalore, Karnataka, India

**Keywords:** Dental anomalies, dens invaginatus, mineral trioxide aggregate, nonsurgical endodontic therapy

## Abstract

The endodontic treatment of teeth with dens invaginatus, characterized by an infolding of enamel and dentin, extending deep into the pulp cavity near the root apex, may be complicated and challenging. The complexity of the internal anatomy may create challenges for the complete removal of diseased pulpal tissue and the subsequent sealing of the canal system. Because of the bizarre root canal anatomy and widely open apex, a combination of nonsurgical and surgical endodontic treatment or extraction is the most common choice of therapy. This article describes case reports of nonsurgical endodontic treatment of Type II dens invaginatus associated with periradicular lesion.

## INTRODUCTION

Dens invaginatus is a developmental malformation of teeth, showing a wide spectrum of anatomical variations. Salter first described anatomical anomaly in 1855 as “a tooth within a tooth.” Ploquet first described it in 1974, which found this malformation in a whale′s tooth. Dens invaginatus is a deep surface invagination of the crown or root that is lined by enamel. Affected teeth show a deep infolding of enamel and dentin starting from the foramen cecum or even the tip of the cusps, which may extend deep into the root.[1] The incidence of dens invaginatus is reported to range from 0.04% to 10%.[2] Maxillary lateral incisors are the most commonly involved teeth. The etiology is controversial and remains unclear; however, most authors conclude that dens invaginatus results from an infolding of the papilla during tooth development.

Oehlers was the first to classify three different types of dens invaginatus according to depth of invagination into the root:[3] Type I: an invagination into the crown only; Type II: an invagination into the root that ends in a blind sac; and Type III: an invagination that penetrates through the root and “bursts” apically or laterally at a foramen, sometimes referred to as a “second foramen” in the root. Dens invagination allows entry of irritants, such as bacteria, into the area that is separated from the pulpal tissue only by a thin layer of enamel and dentin. Therefore, dens invaginatus predisposes to the development of dental caries, usually leading to pulpal necrosis and development of a periradicular lesion.[4] Because of the bizarre root canal anatomy and widely open apex, treatment of teeth with Type 3 dens invaginatus, especially communicating to the periradicular area is more complicated and ranges from nonsurgical root canal treatment[5–9] to surgical treatment[10–12] or extraction.[13] This article describes three case reports of successful nonsurgical endodontic treatment of Type II dens invaginatus and an associated periradicular lesion.

## CASE REPORT

### Case 1

**Figure 1:**
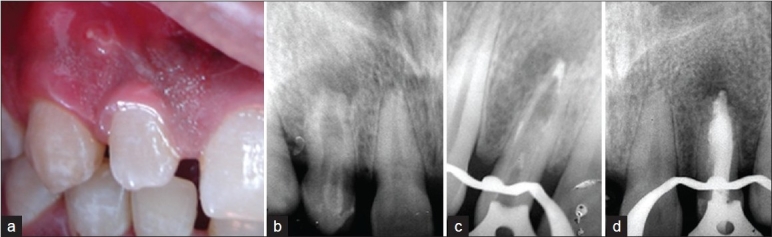
Management of maxillary right lateral incisor with type II dens invaginatus

A 23-year-old male patient reported to Department of Conservative dentistry and Endodontics, V.S. Dental College and Hospital, Bangalore with the complaint of pain and swelling of the gums and subsequent pus discharge from that swelling in the upper right front tooth region of the jaw since a month. A history of recurrent development of gumboil in that region, usually associated with dull-ache was presented. Clinical examination revealed a parulis in the attached gingiva of maxillary right lateral incisor [ Figure 1a]. Tooth was sensitive to percussion and failed to respond to thermal and electrical pulp testing. A periapical radiograph demonstrated maxillary lateral incisor with an Oehlers' type II dens invaginatus, incomplete root end formation and periapical radiolucency [Figure 1b]. A clinical diagnosis of necrotic pulp and chronic periapical abscess was established. Endodontic therapy was performed under rubber dam isolation. Access cavity was prepared and the canal negotiated with small size files. The canal was enlarged and central hard tissue was removed using H-files and Gates Glidden drills with copious irrigation with 5.2% sodium hypochlorite and passive ultrasonic irrigation. Calcium hydroxide dressing was given during the inter-appointment period for 2 weeks. Apical closure was done using mineral trioxide aggregate[14] and tooth was obturated by thermoplasticized technique using Obtura II [Figure 1c and d respectively].

### Case 2

**Figure 2:**
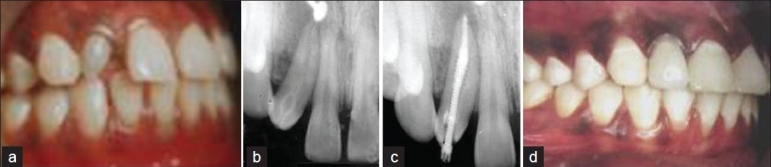
Management of maxillary right lateral incisor with type I dens invaginatus

A 23-year-old female reported to our hospital for endodontic treatment of the maxillary right lateral incisor. The chief complaint of the patient was discolored tooth in relation to maxillary right lateral incisor with a history of recurrent painful swelling over the palatal area for the past 3 years. Medical history was not contributory. Clinical examination showed that both the hard and soft tissues were within normal limits. Maxillary right lateral incisor revealed a yellowish discoloration, distal tilting of clinical crown and a deep depression on lingual surface near the distal marginal ridge [Figure 2d]. This tooth was neither sensitive to percussion and palpation nor responsive to the electric pulp test. On radiographic examination, the right lateral incisor in the midline of this image had an enlarged central cavity and periapical radiolucency was seen [Figure 2a]. A diagnosis of necrotic pulp and chronic apical periodontitis in relation to maxillary right lateral incisor was established. A nonsurgical endodontic therapy was treatment planned.

Under rubber dam isolation, access cavity was prepared. It was found that an invaginated mass of hard tissue almost occupied the whole pulp space. During root canal instrumentation using hand files, a large volume of pale yellow fluid was continuously draining from canal. The canals were irrigated with 5.2% sodium hypochlorite solution along with passive ultrasonic irrigation. Calcium hydroxide (Metapex) was used as an inter-appointment intracanal dressing. The invaginated mass was continuously removed using hand files in combination with copious irrigation. Shaping and cleaning of the canal was completed at the subsequent visit. The tooth was obturated with Gutta percha and root canal sealer using lateral compaction technique. With the evidence of healing, the tooth was restored with post and metal ceramic crown [Figure 2c and d].

### Case 3

**Figure 3:**
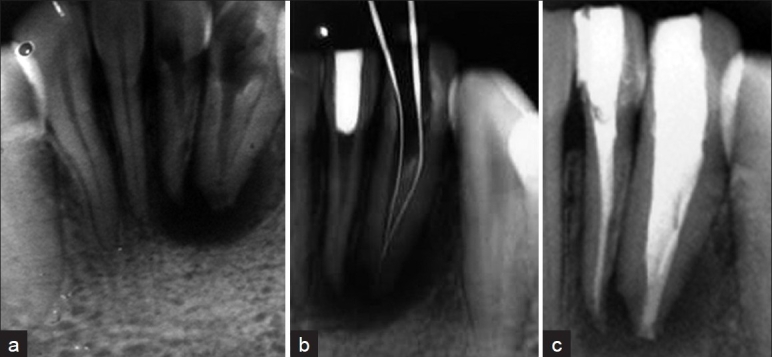
Management of mandibular left lateral incisor with type II dens invaginatus

A 19-year-old girl was referred to our hospital from her village practitioner for the evaluation of a recurrent sinus tract in the lower left lateral incisor region. History revealed recurrent episodes of pain and swelling in that region with subsequent drainage of pus and pain relief since a year. On examination, mandibular left lateral incisor had a comparatively larger crown with an attempted access cavity preparation and an associated draining intraoral sinus. Both teeth were associated with tenderness to percussion and no response to thermal and electrical pulp testing. Periapical radiograph showed a type II dens invagination of lateral incisor and a periapical radiolucency [Figure 3a]. A clinical diagnosis of necrotic pulp and acute exacerbation of a chronic periradicular abscess was established Access cavity was refined in mandibular left lateral incisor and prepared in mandibular left central incisor. Canals were negotiated with small size files. Invaginatus was removed with H-files during shaping and cleaning. 5.2% NaOCl was used along with passive ultrasonic irrigation. Calcium hydroxide (Metapex) was used as an inter-appointment intracanal dressing. This was followed by obturation using Schilder's vertical compaction method. Mandibular left central incisor was endodontically treated and obturated with lateral compaction method. Then, both teeth were restored with composite resin access fillings [Figure 3b and c].

## DISCUSSION

There have been few reports of cases involving endodontic treatment of dens invaginatus. The cases described here demonstrated the anatomical difficulties that can lead to endodontic failure and the development of periapical pathology. Nonsurgical endodontic treatment should be attempted first. Regardless of the size of the periradicular lesion, surgical treatment is the second option only when the nonsurgical endodontic treatment has failed. The successful treatment of this case indicates that the size of the periradicular lesion does not dictate the treatment procedure and influence the treatment outcome of nonsurgical root canal therapy.

Many factors might affect the prognosis of nonsurgical endodontic treatment of this case. First, the severely invaginated central mass of hard tissue almost occupying the whole pulpal space rendered debridement of the canal difficult. Second, the continuous drainage of fluid from the canal during treatment prevented dryness of the canals. Third, the widely open apex negated apical seal of the root canal filling.

Completely debriding the infected canal is the key to the success of this case. The removal of invaginated mass of hard tissue in the canal is difficult and challenging. In this case, it took three visits to totally remove the deeply invaginated mass using hand files.

Calcium hydroxide has been used as interappoinment intracanal medicament.[15] It also has been used to control exudation in the canal.[16] In the above case, we successfully used long-term calcium hydroxide treatment to achieve disinfection.

The use of MTA as an apical seal for immature root allows the immediate rehabilitation of the crown, thus increasing the resistance to fracture and enhancing the esthetic result.[17]

The above case illustrates that even in a tooth with dens invaginatus and an associated large periradicular lesion, nonsurgical endodontic treatment without surgical intervention, can result in satisfactory periradicular healing.

## CONCLUSION

The accurate knowledge of variations in the morphology of pulp cavity will greatly assist the dentist in performing successful endodontic therapy. In cases with complex internal morphology access, cavity refinement may be required for stress-free entry to the root canal system.
